# Improving Adherence Physical Activity with a Smartphone Application Based on Adults with Intellectual Disabilities (APPCOID)

**DOI:** 10.1186/1471-2458-13-1173

**Published:** 2013-12-13

**Authors:** David Pérez-Cruzado, Antonio I Cuesta-Vargas

**Affiliations:** 1 Department of Physiotherapy, University of Malaga, Malaga, Spain; 2 School of Clinical Science, Faculty of Health Science, Queensland University Technology, Queensland, Australia

**Keywords:** Intellectual disability, Physical activity, Adherence, M-Health, Mobile phone, App, IPAQ, Accelerometers

## Abstract

**Background:**

People with intellectual disabilities (ID) have lower levels of physical activity and quality of life and they have a lot of barriers to face when taking part in physical activity. Other problems are the poor adherence to physical activity such people have so this study is designed to improve adherence to physical activity for people with intellectual disabilities with the assistance of an application for smartphones. The aim of the study will be to improve physical activity and physical condition after multimodal intervention and to analyse the promotion of adherence to physical activity through a multimodal intervention and an app intervention (mHealth) in people with ID.

**Methods:**

A two-stage study will be conducted. In stage 1 a multimodal intervention will take place will be done with physical activity and educational advice over eight weeks, two days a week. Data will be measured after and before the intervention. In stage 2 a randomized controlled trial will be conducted. In the intervention group we will install an application to a smartphone; this application will be a reminder to do a physical activity and they have to select whether they have or haven’t done a physical activity every day. This application will be installed for 18 weeks. Data will be measured after and before the application is installed in two groups. We will measure results 10 weeks later when the two groups don’t have the reminder. The principal outcome used to measure the adherence to physical activity will be the International Physical Activity Questionnaire; secondary outcomes will be a fun-fitness test and self-report survey about quality of life, self-efficacy and social support. Samples will be randomized by sealed envelope in two groups, with approximately 20 subjects in each group. It’s important to know that the therapist will be blinded and won’t know the subjects of each group.

**Discussion:**

Offering people with ID a multimodal intervention and tool to increase the adherence to a physical activity may increase the levels of physical activity and quality of life. Such a scheme, if beneficial, could be implemented successfully within public health sense.

**Trial registration:**

ClinicalTrials.gov Identifier: NCT01915381.

## Background

About 1.5% of the adult population have intellectual disabilities (ID), and in over half of these cases the cause of this problem is unknown and this number is higher in developing countries [1]. People with ID have a low level of healthy lifestyle, poor dietary habits and low physical activity (PA). The latter may be due to the number of barriers they face in doing a physical activity and to the few resources and opportunities open to them [2]. However, currently awareness of the barriers and facilitators for people with ID which enable them to take part in physical activity is poor [3] so we find health problems like obesity or chronic disease in this population [4]. People with ID have a poor maintenance in physical activity and it’s important to try to help them to improve this maintenance [5]. In people with ID we find lower levels of physical fitness compared with the general population [6].

Physical activity provides many benefits to people with intellectual disabilities [7] helping to reduce the risk of hypertension, coronary heart disease, stroke, diabetes, breast cancer and colon cancer, depression and falls, and is a key to energy balance and weight control [8]. Most studies use physical intervention to improve physical activity and physical condition, but it is critical that this intervention will be a multimodal intervention (intervention with physical activity and educational advice) [9]. In the bibliography we only found studies that try to improve adherence to the physical activity, but these studies only use physical intervention, not multimodal intervention with educational advice. Most studies use physical intervention to improve physical activity and physical condition, but it is critical that this intervention is a multimodal intervention (an intervention that combines PA and educational advice) [9]. This study should be relevant, because in our opinion this study is the first long-term study to promote PA in people with ID that uses a multimodal intervention, and other authors confirm that studies to promote PA in people with ID are needed because these people face many barriers when they want to practice PA [10,11]. Another problem that we have found is the poor adherence to PA [5], because there are few studies that measure long-term adherence. As a consequence, in our study we expect to improve this adherence through an application for mobile phones where people with ID will have a reminder every day, and results will be measured to ascertain whether a reminder can change the outcome and increase adherence. Nowadays, there are studies that use TICs to improve adherence to a treatment by patients and the results of these studies have been positive [12,13]. We also found a study similar to ours, but the sample studied wasn’t of people with ID; in this study they used an app versus traditional methods to compare self-monitoring in physical activity [14]. To our knowledge there isn’t an app to improve adherence to PA in people with ID, however, an app development for this, and tested in an RCT, may determine whether the expected effect of the multimodal intervention improves adherence with the assistance of the app. The present study will have two aims: the first is to improve physical activity in people with ID after multimodal intervention, and the second is the promotion of adherence to physical activity through an app intervention (mHealth) for people with ID. The hypothesis of our study is to check whether the reminder from the smartphone improves adherence to physical activity in people with ID after multimodal intervention.

## Method/Design

### Design

A two-stage study will be conducted. In stage 1 a prospective study will be conducted. Data will be measured after and before the intervention. In stage 2 a randomized controlled trial will be conducted.

### Participants

Forty people with a mild to moderate level of ID and ranging in age from 18 to 65 will be recruited from an occupational centre (ASPROMANIS, Malaga, Spain). They must be able to read and write in order to answer the questions, they need to be capable of installing the reminder on their smartphone, and they must not be suffering from any disease that will prevent them from undertaking a physical activity.

Before starting the investigation we have guaranteed participants the protection of confidential information obtained from them [Law 15/1999 Protection of Personal Data]. Informed consent was obtained from all subjects, and study procedures were consistent with the Helsinki declaration. The Faculty of Health Sciences Research Committee approved this protocol (UMA-01-13).

### Procedure

**Figure 1:**
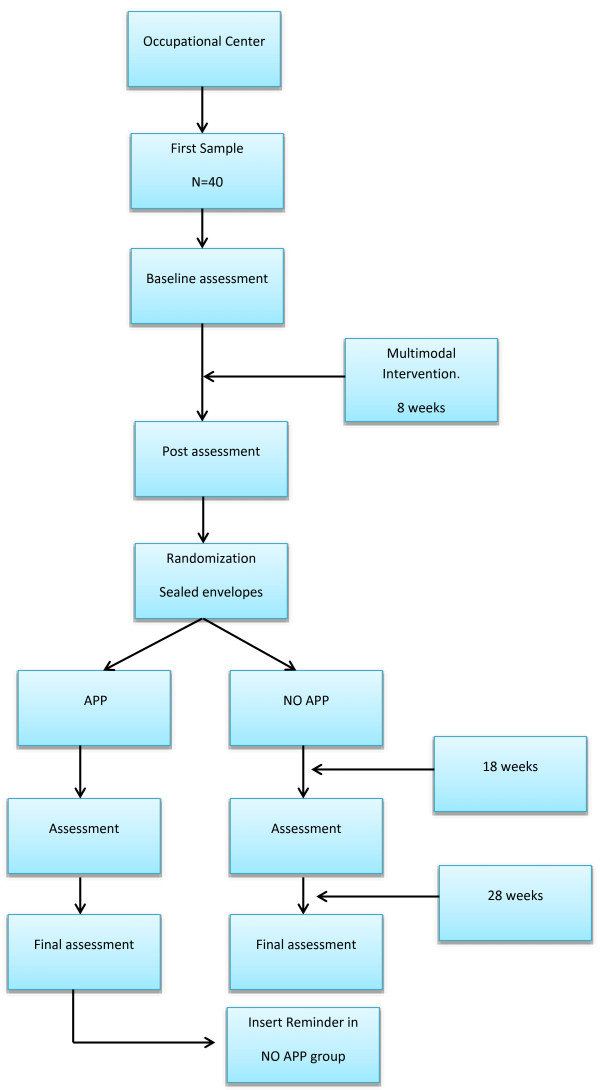
Flow chart of the study.

For multimodal intervention (stage 1), we have to divide the sample into three working groups, with around 13 people in each group for a low ratio. This intervention will be carried out over eight weeks, twice a week. For the reminder app intervention (stage 2), we will divide the sample into two groups, the assignment of subjects in each group is through a system of sealed envelopes; in this way we will have one group that will have the app reminder on their smartphone and another group that will not. The drop-out of participants in stage 2 will be counted (Figure 1).

### Intervention

All subjects receive a multimodal intervention consisting of a program with an educational approach and physical activity, where a therapist advises on the benefits of PA [15]. Multimodal intervention will be carried out in ASPROMANIS 2 hours weekly over eight weeks. In each session the therapist will provide educational advice brochure [15] with the physical activity to improve strength, flexibility, balance and aerobic condition. Variables will be measured before and after intervention.

Below we separate the sample into two groups, where one group have a reminder on their smartphone and another group are the control group; a researcher by means of a sealed envelope will do assignment to the groups; the therapist will be blinded and will not know what subjects are in each group. The smartphone-based application group will have a reminder find out whether they have done PA every day; in this app this group will have to select whether they have or haven’t done a physical activity.

After 18 weeks, results will be measured to ascertain the adherence in the smartphone group (SG) and the no-smartphone group (NSG, and we will learn whether the reminder has realized the expected effect by comparing the results between both groups. Ten weeks after the reminder has been removed, we will measure the results in the SG and the NSG again, to establish whether the SG continue their adherence to PA and compare this to the NSG.

### Outcome measures

Summary of tools and variables of the study are shown in the Table 1.

**Table 1:** Summary of tools and variables of the study

**Name**	**Measure**	**Variable type**
**IPAQ**	Physical Activity	Main
**Accelerometer Gt3x**	Physical Activity	Secondary
**WHOQoL-DIS**	Quality of life	Secondary
**Barthel**	Level of dependence	Secondary
**FunFitness**	Physical Condition	Secondary
**SE/SS AID**	Social support and autoefficacy	Secondary
**Raven´s progressive matrices**	IQ	Secondary
**Open Interview**	Barriers and facilitators to physical activity	Secondary

#### Primary outcome measure: adherence to PA

In this study the principal outcome is the adherence to PA and will be measured using the *International Physical Activity Questionnaire (IPAQ)*[16] this is a good scale to measure physical activity. With the IPAQ scale we will learn the time spent in vigorous and moderate physical activity in the last seven days and the time spent walking or sitting in the last seven days (https://sites.google.com/site/theipaq/). We use IPAQ-Short Version because it is easier for people with ID to understand and there are studies where this scale is used to measure physical activity in people with ID [17,18]. In each session the therapist will provide educational advice from the advice list [15] with the physical activity to improve strength, flexibility, balance and aerobic condition. The principal outcome to measure the adherence is IPAQ because with data obtained after and before the reminder from the IPAQ we will learn whether we have achieved the expected effect using the reminder; if we obtain better results in IPAQ, we can affirm that the reminder in the smartphone improves adherence. Reliability of the original version was Kappa coefficient = 0.6 [19].

#### Secondary outcome measures

We will use accelerometers to measure PA too, because *accelerometers* have previously been found to reliably measure the PA of adults with ID [20]. In our study we will use Actigraph GT3X. A selected group of participants will wear Actigraph for a week to measure PA and this will provide us with more information about the PA of that sample.

To measure physical condition we also to review the *Fun*-*fitness programme test*, Special Olympics; with this programme we can learn about the physical condition of the patients and help them with suggestions on how to improve their physical condition and to avoid injuries. When this test is performed, all participants receive information about their physical profile, and recommendations on how to increase their physical qualities. (http://www.specialolympics.org). In our study we will measure 13 items which cover strength, aerobic condition, balance and flexibility in the sample –for more details see Cuesta-Vargas et al. 2011 [21]. There is also another scale to establish physical condition: this is the FunFitness battery test with nine physical test, developed in another study by Cuesta-Vargas et al. 2013 [22], but we will use the 13 items FunFitness scale because it is fuller.

*Self-report outcome measures* will be used to measure quality of life using the *WHOQoL scale*[23] because this is the scale most recommended by WHO to measure QoL, and because the sample of our study are people with ID, and in this scale they have to answer 12 items in which there are only three possible answers (1, 2 and 3). This scale has also been used in other studies to measure QoL in people with ID in Spain [24]. Reliability of the original version of WHOQol scale was α Cronbach 0.90 [25]. To measure the level of dependence we will use the *Barthel index*; this index provides us quantitative information about the level of dependence, measuring the execution of ten daily life activities [26] (α Cronbach 0.86-0.92) [26]. For social support and self-efficacy for leisure activity we will use the self-efficacy/social support for activity for persons with intellectual disability scale (SE/SS-AID) [27]. In this scale the sample have to answer six self-efficacy items and 17 social support items with three possible answers. Reliability of SE/SS AID is (α Cronbach 0.70-0.74) [28]. We will use a questionnaire about socio-demographic data, with gender, age, level of study, birthplace and associated diseases.

### Statistical treatment

Mean and standard deviations or 95% confidence intervals of the values will be calculated for each variable. Pre-intervention values prior to each condition will be compared using the independent t-tests for continuous data. A 2x2 mixed model ANOVA with supplementation (SG or NSG) as the between-subjects variable and time (pre-; post-intervention) as the within-subjects variable will be used. The hypothesis of interest was intervention * time interaction. The Bonferroni test will be used for post hoc analysis. A P-value < 0.05 was considered statistically significant. Data will be analysed using the SPSS package (version 19.0).

### Sample size calculation

A priori sample size calculation for stage 2 indicated 15 patients per group were required in order to detect a significant difference of 25% in IPAQ [29] between the intervention and control group (Effect size d = 1, alpha = 0.05, beta = 0.08).

## Discussion

If offering people with ID a multimodal intervention and tool to increase adherence to PA increases the levels of physical activity and quality of life, such a scheme, if beneficial, could be implemented successfully within public health policies.

In stage 1, this study will use a multimodal intervention with physical activity and an educational approach to establish whether this intervention changes the physical activity of people with ID. There are many studies that use physical intervention to improve physical activity, but in the bibliography there are no studies that use educational advice with physical activity, so to our knowledge this is the first study to use a multimodal intervention to improve physical activity in people with intellectual disabilities.

In stage 2 this study will investigate the effectiveness of an app (reminder) in a smartphone to improve adherence to physical activity, contrasting results between the smartphone and the no-smartphone groups. The use of an app to improve the physical activity of people with intellectual disability is an innovative method. The strength of this study is, to our knowledge, that it is the first that will use an app to improve adherence to physical activity in people with intellectual disabilities; there are other studies that use an app to improve adherence in physical activity but with a different population sample [30]. Therefore, the results of this study will be very helpful in applying the use an app to people with ID. Finally this study will introduce new technology to people with intellectual disabilities, and so this is a first step for this population to use mHealth.

## Abbreviations

RCT: Randomized controlled trial; ID: Intellectual disabilities; GCP: Good clinical practice; PA: Physical activity; SG: Smartphone group; NSG: No-smartphone group; IPAQ: International Physical Activity Questionnaire; WHOQoL: World Health Organization Quality of Life; IC: Intellectual coefficient; App : Application.

## Competing interests

The authors declare that they have no competing interests.

## Authors’ contributions

AIC-V has made a contribution to the conception of this study. DP-C and AIC-V drafted the protocol and manuscript. Both authors have given final approval of the version to be published.

## Pre-publication history

The pre-publication history for this paper can be accessed here:

http://www.biomedcentral.com/1471-2458/13/1173/prepub
